# Evolution of Helium Bubbles and Discs in Irradiated 6H-SiC during Post-Implantation Annealing

**DOI:** 10.3390/ma10020101

**Published:** 2017-01-24

**Authors:** Qiang Shen, Wei Zhou, Guang Ran, Ruixiang Li, Qijie Feng, Ning Li

**Affiliations:** 1 College of Energy, Xiamen University, Xiamen 361102, Fujian, China; shenqiang1989@126.com (Q.S.); xiangruimantian@126.com (R.L.); ningli@xmu.edu.cn (N.L.); 2 China Academy of Engineering Physics, Mianyang 621900, Sichuan, China; zhouwei_801202@163.com (W.Z.); fengqijie@caep.cn (Q.F.)

**Keywords:** SiC, irradiation, annealing, bubble growth, microstructure

## Abstract

The single crystal 6H-SiC with [0001] crystal direction irradiated by 400 keV He^+^ ions with 1 × 10^17^ ions/cm^2^ fluence at 400 °C were annealed at 600, 900, 1200 and 1400 °C for different durations. The evolution of helium bubbles and discs was investigated by transmission electron microscopy. An irradiated layer distributed with fine helium bubbles was formed with a width of ~170 nm after helium ion irradiation. The size of gas bubbles increased with increasing annealing time and temperature and finally reached stable values at a given annealing temperature. According to the relationship between the bubble radii and annealing time, an empirical formula for calculating the bubble radii at the annealing temperature ranged from 600 to 1400 °C was given by fitting the experiment data. Planar bubble clusters (discs) were found to form on (0001) crystal plane at both sides of the bubble layer when the annealing temperature was at the range of 800–1200 °C. The mechanism of bubble growth during post-implantation annealing and the formation of bubble discs were also analyzed and discussed.

## 1. Introduction

There have been considerable efforts devoted to the development of SiC for nuclear energy applications by utilizing its unique physical and chemical properties. These properties include excellent mechanical and chemical stability at high temperature and low neutron capture cross-section, making SiC a leading candidate material suitable for high temperature, high irradiation and extreme environmental conditions [1,2]. SiC has been used as the middle layer for preventing fission product diffusion and providing structural support in TRISO fuel particles [3,4]. In recent accident tolerant fuel (ATF) research projects, SiC/SiC composite materials are regarded as safe fuel cladding [5,6]. However, the transmutation reaction (n, α) generates helium atoms that migrate, gather and grow in SiC to form gas bubbles, which degrade the service performance [7]. The existent forms of helium atoms in SiC include isolated atoms, dislocation loops, bubbles, discs and platelets, which induce performance degradation such as hardening, embrittlement and irradiation swelling [8,9]. Therefore, it is important to investigate the evolution of helium in SiC.

Although the microstructure evolution of SiC irradiated by helium ions has been widely investigated in the past twenty years [9,10,11,12,13,14,15,16], there are still some questions that need to be addressed and answered, such as the detailed evolution of bubbles and discs. Zhang et al. [9] investigated the dependence of helium-defect formation with ion fluence in 4H-SiC. High density of bubbles in alignment was formed in the basal plane after implantation with 10^17^ ions/cm^2^ fluence. Chen [10] reported the bubble growth with annealing time at 1720 K. The growth behavior and evolution mechanism of helium bubbles should be systematically investigated with different annealing temperature and time. Wei Hua [11] reported the bubble discs only formed at the sides of irradiated region while in the middle of irradiated region there were isolated bubbles with a fluence of 10^17^ ions/cm^2^. A model was provided to explain such sandwich distribution of helium defects by Zhang [12]. However, Chen [13] observed the formation of helium platelets with a uniform diameter of ~9 nm and thickness of 0.6 nm after 2450 appm helium implantation throughout the whole irradiated region. The morphology of platelets kept stable upon annealing up to 1270 K. At 1273 K annealing temperature, Li [14] observed the formation of bubble discs in the irradiated area of 6H-SiC irradiated with about 3 × 10^16^ He^+^/cm^2^ fluence. Strain induced by helium implantation in SiC crystal also affected the evolution of irradiation defects during ion implantation and subsequent annealing [15]. However, the detailed evolution behavior of bubble discs has not been fully investigated during post-implantation annealing.

In the present work, the evolution and mechanism of helium bubbles and discs in single crystal 6H-SiC irradiated with 1 × 10^17^ ions/cm^2^ fluence were investigated during the annealing process. The relationships between average bubble sizes and annealing parameters such as temperature and time were also analyzed.

## 2. Experiments

The single crystal 6H-SiC with [0001] crystal direction from MTI Corporation were irradiated by 400 keV helium ions with a fluence of 1 × 10^17^ ions/cm^2^. The ion implantation was performed at 400 °C to avoid amorphous transformation of 6H-SiC. The irradiated samples were subsequently annealed at 600 °C, 900 °C, 1200 °C and 1400 °C for different durations under argon atmosphere. According to the simulation results of SRIM 2008 software (Quick mode, SRIM.EXE, (C) 1984-2013, James F. Ziegler, Annapolis, MD, USA), helium implantation with a fluence of 1 × 10^17^ ions/cm^2^ caused a peak helium concentration of 5.8% at the depth of 1.15 μm. The peak displacement damage was about 2.5 dpa at the depth of 1.08 μm.

Cross-sectional transmission electron microscopy (TEM) samples were prepared by a method of mechanical thinning and then ion milling. The SiC bulk was first glued to single crystal Si substrate by G-1 epoxy glue. The SiC-glue-Si sandwich was cut into the slices with approximate 0.5 mm thickness along with the direction of ion incident and then mechanically polished to approximate 15 μm thickness by diamond sandpapers. Then the thinned flake was glued on a copper grid by G-1 epoxy glue and finally thinned to about 100 nm thickness via Ar ion milling by Gatan 695 PIPS (Gatan, Inc., Pleasanton, CA, USA). The microstructure of cross-sectional TEM samples was observed by TEM on a JEOL-2100 instrument (JEOL, Tokyo, Japan). According to the obtained TEM images, the distribution characteristics of gas bubbles could be obtained by Photoshop software. The size of gas bubbles were measured by the function of Particle Analysis in an ImageJ software. The average size of gas bubbles (μ) and the standard deviation (σ) were calculated.

## 3. Results and Discussion

**Figure 1:**
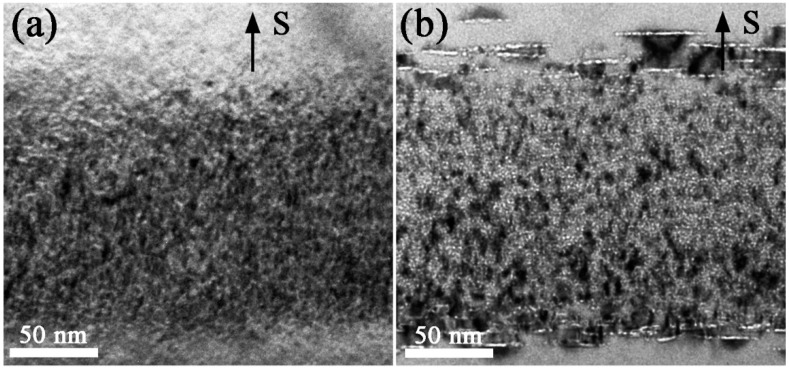
Bright field TEM images showing the 6H-SiC samples implanted with 1 × 10^17^ ions/cm^2^ fluence at 400 °C: (**a**) as-implanted; (**b**) annealed at 900 °C for 30 min. The letter “S” and black arrows indicate the irradiation surface.

Figure 1 shows the TEM images of 6H-SiC samples irradiated by helium ions with 1 × 10^17^ He^+^/cm^2^ fluence at 400 °C and then annealed for 30 min at 900 °C. An irradiated layer with heavy damage contrast is observed in the as-irradiated sample as shown in Figure 1a. A large number of tiny helium bubbles with an average radius 0.3 nm distribute uniformly in the irradiated layer. The lattice damages are significantly recovered while the increased size of helium bubbles is very small after annealing for 30 min at 600 °C. When the annealing temperature is at 900 °C and 1200 °C, a sandwich structure is formed in the irradiation layer, where the planar bubble clusters (also named discs) are formed on (0001) crystal plane at both sides of bubble layer as shown in Figure 1b. The discs are surrounded by strong strain described as butterfly wings [17]. The bubble discs can be observed after only annealing for five minutes at 900 °C. The size of gas bubbles in the bubble discs is always larger than that of gas bubbles in the bubble layer. However, no discs can be observed when the annealing temperature is above 1400 °C.

**Figure 2:**
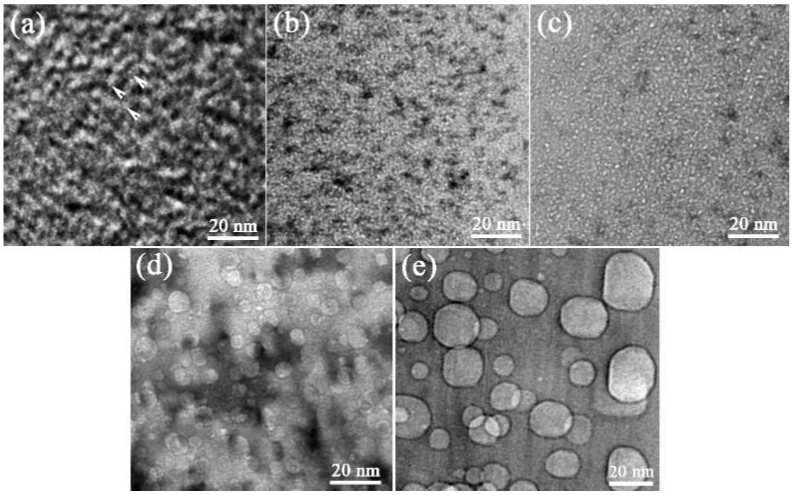
Bright field TEM images showing the morphology of helium bubbles in the SiC samples irradiated with 1 × 10^17^ ions/cm^2^ fluence at 400 °C: (**a**) as-implanted; (**b**–**e**) annealed for 15 h at 600 °C, 900 °C, 1200 °C and 1400 °C. TEM images were taken at the under-focused condition of 300 nm.

**Figure 3:**
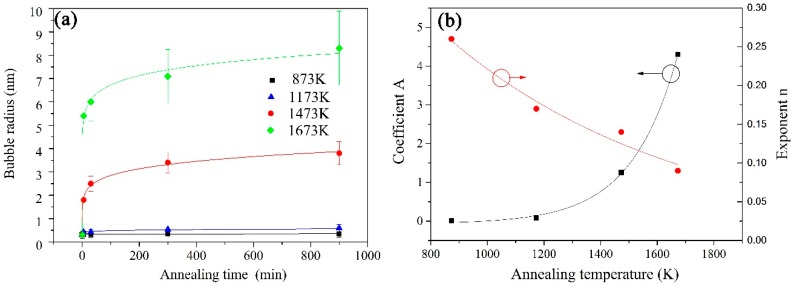
Curves showing the growth behavior of gas bubbles during the post-implantation annealing: (**a**) bubble sizes vs. annealing time; (**b**) coefficient *A* and exponent *n* vs. annealing temperature *T*.

Figure 2 shows the gas bubbles in the as-implanted and post-implantation samples annealed for 15 h at 600 °C, 900 °C, 1200 °C and 1400 °C. The evolution of gas bubbles is significantly affected by the annealing temperature and time. The gas bubbles uniformly distribute in the irradiated layer. The bubble size shows the independence of helium concentration gradient during the annealing process. The average size of bubbles increases with increasing annealing temperature. Figure 3a shows the relationship between the average radii of helium bubbles and annealing time at four annealing temperatures. The average radii were calculated by Gaussian fitting and the error bar value is the standard deviation of Gaussian fitting. The average radius of helium bubbles increases from 0.3 nm at the as-irradiated state to 0.6 nm after annealing for 15 h at 900 °C. The growth rate is slow at 900 °C annealing. However, the bubbles grow noticeably when the annealing temperature is above 1200 °C. After annealing for 15 h, the average radius increases from 0.3 nm at the as-irradiated state to 3.6 nm at 1200 °C and to 8.3 nm at 1400 °C. According to the experiment data in Figure 3a, the relationship between helium bubble radius and annealing time at a given annealing temperature is satisfied with power law and can be fitted by following equation:

(1) $$R_{b}=A\times t^{n}+R_{0}$$

where, *R_b_* is the mean radius of gas bubbles in nanometers and the subscript *b* represents the specific temperature; *t* is annealing time in minutes; and *R*_0_ = 0.3 nm is the original bubble radius after helium implantation at 400 °C. The coefficient *A* and exponent *n* are temperature-dependent as shown in Figure 3b. The value of *A* and *n* can be calculated as follows:

(2) $$A=1.7\times 10^{-4}\cdot e^{T/165}-0.07$$

(3) $$n=0.7\cdot e^{-T/840}-0.2$$

The Equations (1)–(3) can be used to approximately calculate the helium bubble radii in the irradiated 6H-SiC annealed at a specified temperature and time.

**Figure 4:**
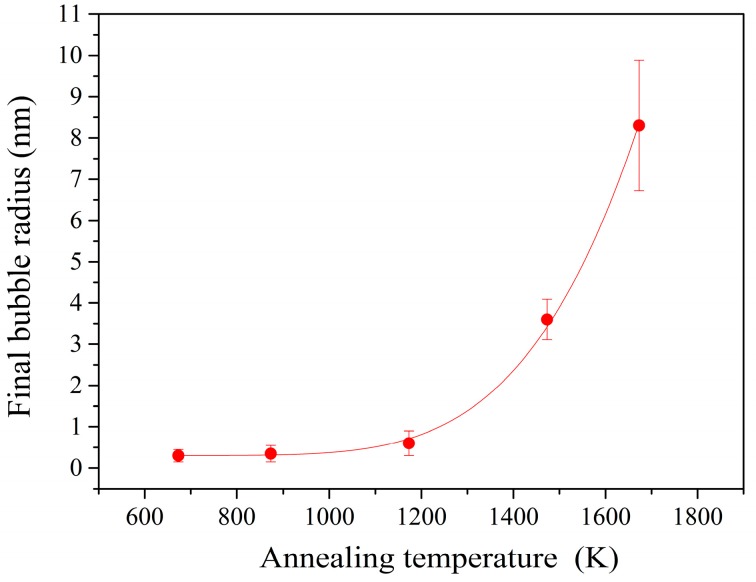
The relationship between final bubble radius and annealing temperature.

The bubbles tend to stop growth after 15 h of annealing and reach a constant value at a given annealing temperature. Certainly, the final sizes of gas bubbles are related to annealing temperature. From Figure 4, the relationship between the final radius *R_T_* of gas bubbles after annealing at a given temperature *T* and the effective activation energy of coarsening *E*_r_ can be expressed as the equation below:

(4) $$R_{T}=A_{0}\cdot \exp\left(-\frac{E_{r}}{kT}\right)+R_{0}$$

where, *k* is Boltzmann constant; *E*_r_ is the effective activation energy of coarsening; *T* is annealing temperature; and *A*_0_ is the coefficient. The *A*_0_ and *E*_r_ are calculated to be 8640 and 1.01 eV. The fitting formula can be used to estimate the final bubble radii in the irradiated 6H-SiC after sufficient annealing above 400 °C with the irradiation fluence of 1 × 10^17^ He^+^/cm^2^.

During the post-implantation annealing, the average size of bubbles increases with increasing annealing time and the bubble density drops down. The width of the bubble layer does not broaden and no visible bubbles are observed outside the bubble discs. Therefore, only attention to bubble coarsening in the bubble layer is needed. It is assumed the proportion of helium atoms escaping from the bubble layer is small and negligible (smaller than 20% reported in Jiang’s work [18]). There are mainly two mechanisms used to explain bubble coarsening in the irradiated materials: bubble migration and coalescence (MC) and Ostwald ripening (OR) [19,20,21]. MC is always controlled either by surface diffusion (MC/S) or by volume diffusion (MC/V). OR is considered to be a more complicated process. The actual process includes the dissociation of helium and vacancies from small bubbles, the diffusion of helium atoms and vacancies, and the re-absorption of helium atoms by large bubbles. OR of bubble growth can be simplified in two ways: controlled by gas (He) dissociation and vacancy dissociation [19].

The calculation of the value of *n* = ∂ln*r*/∂ln*t* based on Equation (4) could be one of the criteria to distinguish bubble growth mechanisms [20]. The values *n* are 1/6–1/3 for MC and <1/2 for OR. The effective radius “*r*” in equation is modified by subtracting the original radius 0.3 nm formed during the helium implantation at 400 °C. So, the values of *n* = 0.26, 0.17, 0.15 and 0.09 at 600 °C, 900 °C, 1200 °C and 1400 °C annealing are derived from the modified calculation formula *n* = ∂ln(*R*_b_ − *R*_0_)/∂ln*t*, respectively. Judging from the *n* value calculation, both MC and OR mechanisms can be used to explain the bubble coarsening behaviors. However, Trinkaus et al. suggested that the *n* value cannot be a fully reliable criterion [19].

According to Chernikov’s work [21], $E_{\mathrm{He}}^{\mathrm{diss}}$, $E_{V}^{\mathrm{diss}}$ are equal to 2Ễ_r_ and 3Ễ_r_, respectively. $E_{\mathrm{He}}^{\mathrm{diss}}$, $E_{V}^{\mathrm{diss}}$ and Ễ_r_ are the mean activation energy of helium dissociation from defects, the mean activation energy of vacancy dissociation from a bubble and the theoretical effective activation energy of bubble coarsening, respectively. $E_{V}^{\mathrm{diss}}$ is approximately equal to the activation energy of the self-diffusion, *E*_V_. The activation energies calculated from our work could be written as follows: $E_{V}^{\mathrm{diss}}$ = 2  Ễ_r_ = 2.02 eV, $E_{V}^{\mathrm{diss}}$ = 3 Ễ_r_ = 3.03 eV, respectively. Pramono reported that helium atoms diffuse via a dissociative/interstitial mechanism with small diffusion activation energy of $E_{\mathrm{He}}^{\mathrm{diss}}$ = 0.9 eV [22]. So the activation energy of helium dissociation is significantly smaller than the calculated one in our work. Thus the dissociation of helium atoms could be easily achieved during the annealing. The *E*_V_ of Si vacancy and C vacancy are reported to be 3.2–3.6 eV [23] and 3.5–4.1 eV, respectively [24]. The value is close to the effective activation energy $E_{V}^{\mathrm{diss}}$ = 3.03 eV calculated from our experiment data. Therefore, this indicates that the bubble growth is controlled by vacancy dissociation of OR. Another hint is that the pressure of helium bubbles in SiC is extremely high (approximate 24 Gpa) [12]. Trinkaus’ paper showed that the vacancy dissociation of OR dominated during the bubble growth with high internal bubble pressure [25].

The diffusion activation energies of Si interstitials and C interstitials are 0.74 eV and 1.53 eV, respectively [24]. Both helium atoms and Si/C interstitials can migrate at low temperature (~600 °C) while the mobility of Si/C vacancies is limited [26,27]. When annealed at low temperatures (~600 °C), the damaged regions are recovered via the recombination of close Frenkel pairs. When it is annealed at elevated temperatures >800 °C [28,29], long-range migration of vacancies takes place. The migration of vacancies is reported to be accelerated above 1200 °C [27]. Therefore, the growth behavior of helium bubbles in the present work can be well explained, which includes very tiny growth at 600 °C, the limited coarsening at 900 °C and the rapid growth at 1200 °C and 1400 °C.

It is very interesting that the fine bubbles uniformly distributed in a thickness of 170 nm as shown in Figure 2, while in that depth range SRIM simulation results show a normal distribution of helium concentration and displacement damage for SiC irradiated with 1 × 10^17^ He^+^/cm^2^ fluence. When the irradiation damage exceeded a threshold value, the lattice disorder reached a saturation level at the damage peak region (the disorder level of 1.0 corresponds to fully amorphization) [18]. The relative lattice disorder saturation level is obviously related to the implantation temperature. The lattice disorder saturation value is relatively lower at 400 °C than that at RT. Thus a saturated lattice disorder layer with a lower value was formed in the highly damaged region at 400 °C. Helium-vacancies clusters started to form equivalently in this saturated lattice disorder layer, which acted as bubble nuclei [29]. The bubbles in the bubble layer behaved the same during the annealing. This is quite different with room-temperature helium implantation of SiC in Li’s work [30]. After annealing of the RT helium implantation, the distribution of bubble sizes is proportionate to the damage (helium concentration) distribution [30]. That is because the irradiated layer became totally amorphous after implantation. The recrystallization of amorphous layer and bubble growth took place simultaneously during the annealing. The bubble coarsened via gathered helium atoms and vacancies with no need to expel neighboring lattice atoms. Thus the helium concentration peak region has the biggest bubbles.

In the present work, when annealed at low temperatures (600 °C), no bubble discs were formed at the boundaries of bubble layer and SiC substrate. Bubble discs were formed at two boundaries when annealed at 900 °C and 1200 °C. Leclerc et al. also observed bubble discs at 800 °C and 1400 °C [31]. However, spherical bubbles but no bubble discs were formed when annealed at 1400 °C in this work. It is safely concluded that the bubble discs can be formed when annealed in the temperature range of 800–1200°C. Firstly, it is believed a helium fluence threshold is essential for the formation of sandwich structure [9]. Then the formation of discs at the boundaries are related to the motion of vacancies and interstitials. The vacancies started to migrate above 900 °C. During the annealing, interstitials in the near surface region drifted back into highly strain region (the irradiated layer verified by SRIM calculation) [15]. Oliviero’s model also suggested self-interstitials returned to the peak irradiation region from two sides of the irradiated layer during annealing [32]. The interface region of the bubble layer and SiC substrates experienced release of lattice strain and decrease of interstitials with lower helium concentration. Then the boundaries become effective sinks for vacancies. The vacancies and helium atoms can readily diffuse to the interfaces to form bubbles. The average radius of bubbles inside the bubble discs, which is bigger than that of bubbles in the irradiated layer, well supported this process. The average radii of bubbles inside the bubble discs and in the irradiated layer are 1.3 nm and 0.6 nm when annealed at 900 °C for 30 min. Leclerc’s study showed the relaxation of strain was mainly along the implanted direction [33]. The strain was released along [0001] direction in his work. This could explain that the bubble discs are mainly formed on (0001) planes.

## 4. Conclusions

The microstructure of single crystal 6H-SiC with [0001] crystal direction irradiated by 400 keV He^+^ ions with 1 × 10^17^ ions/cm^2^ fluence at 400 °C and subsequently annealed at 600, 900, 1200 and 1400 °C for different durations was analyzed by transmission electron microscopy. An irradiated layer with a large number of tiny bubbles was formed with a width of 170 nm. The size of gas bubbles increased with increasing annealing time and temperature. The mean radius of bubbles finally reached a stable value at a given annealing temperature. An empirical formula of bubble radius and annealing time was obtained from the experiment data, which could be used to calculate the helium bubble sizes at a given annealing temperature in 6H-SiC. Bubble coarsening was mainly controlled by vacancy dissociation of OR mechanism. Bubble discs lying on (0001) crystal plane were formed at both sides of the bubble layer when the annealing temperature ranged from 800 to 1200 °C. The formation of discs at the boundaries of the bubble layer was attributed to the migration of self-interstitials and the accumulation of helium-vacancy clusters at the boundaries induced by thermal annealing.
